# Mitochondrial Voltage-Dependent Anion Channel Protein Por1 Positively Regulates the Nuclear Localization of *Saccharomyces cerevisiae* AMP-Activated Protein Kinase

**DOI:** 10.1128/mSphere.00482-17

**Published:** 2018-01-10

**Authors:** Aishwarya Shevade, Vera Strogolova, Marianna Orlova, Chay Teng Yeo, Sergei Kuchin

**Affiliations:** a Department of Biological Sciences, University of Wisconsin—Milwaukee, Milwaukee, Wisconsin, USA; University College Dublin, Belfield

**Keywords:** AMPK/Snf1, VDAC, mitochondria, nuclear localization, yeast

## Abstract

Snf1 protein kinase of the yeast *Saccharomyces cerevisiae* is a member of the highly conserved eukaryotic AMP-activated protein kinase (AMPK) family, which is involved in regulating responses to energy limitation. Under conditions of carbon/energy stress, such as during glucose depletion, Snf1 is catalytically activated and enriched in the nucleus to regulate transcription. Snf1 catalytic activation requires phosphorylation of its conserved activation loop threonine (Thr210) by upstream kinases. Catalytic activation is also a prerequisite for Snf1’s subsequent nuclear enrichment, a process that is mediated by Gal83, one of three alternate β-subunits of the Snf1 kinase complex. We previously reported that the mitochondrial voltage-dependent anion channel (VDAC) proteins Por1 and Por2 play redundant roles in promoting Snf1 catalytic activation by Thr210 phosphorylation. Here, we show that the *por1*Δ mutation alone, which by itself does not affect Snf1 Thr210 phosphorylation, causes defects in Snf1 and Gal83 nuclear enrichment and Snf1’s ability to stimulate transcription. We present evidence that Por1 promotes Snf1 nuclear enrichment by promoting the nuclear enrichment of Gal83. Overexpression of Por2, which is not believed to have channel activity, can suppress the localization and transcription activation defects of the *por1*Δ mutant, suggesting that the regulatory role played by Por1 is separable from its channel function. Thus, our findings expand the positive roles of the yeast VDACs in carbon/energy stress signaling upstream of Snf1. Since AMPK/Snf1 and VDAC proteins are conserved in evolution, our findings in yeast may have implications for AMPK regulation in other eukaryotes, including humans.

**IMPORTANCE** AMP-activated protein kinases (AMPKs) sense energy limitation and regulate transcription and metabolism in eukaryotes from yeast to humans. In mammals, AMPK responds to increased AMP-to-ATP or ADP-to-ATP ratios and is implicated in diabetes, heart disease, and cancer. Mitochondria produce ATP and are generally thought to downregulate AMPK. Indeed, some antidiabetic drugs activate AMPK by affecting mitochondrial respiration. ATP release from mitochondria is mediated by evolutionarily conserved proteins known as voltage-dependent anion channels (VDACs). One would therefore expect VDACs to serve as negative regulators of AMPK. However, our experiments in yeast reveal the existence of an opposite relationship. We previously showed that *Saccharomyces cerevisiae* VDACs Por1 and Por2 positively regulate AMPK/Snf1 catalytic activation. Here, we show that Por1 also plays an important role in promoting AMPK/Snf1 nuclear localization. Our counterintuitive findings could inform research in areas ranging from diabetes to cancer to fungal pathogenesis.

## Abstract (precis)

AMP-activated protein kinases (AMPKs) sense energy limitation and regulate transcription and metabolism in eukaryotes from yeast to humans. In mammals, AMPK responds to increased AMP-to-ATP or ADP-to-ATP ratios and is implicated in diabetes, heart disease, and cancer. Mitochondria produce ATP and are generally thought to downregulate AMPK. Indeed, some antidiabetic drugs activate AMPK by affecting mitochondrial respiration. ATP release from mitochondria is mediated by evolutionarily conserved proteins known as voltage-dependent anion channels (VDACs). One would therefore expect VDACs to serve as negative regulators of AMPK. However, our experiments in yeast reveal the existence of an opposite relationship. We previously showed that *Saccharomyces cerevisiae* VDACs Por1 and Por2 positively regulate AMPK/Snf1 catalytic activation. Here, we show that Por1 also plays an important role in promoting AMPK/Snf1 nuclear localization. Our counterintuitive findings could inform research in areas ranging from diabetes to cancer to fungal pathogenesis.

## INTRODUCTION

The AMP-activated protein kinase (AMPK) family is conserved in eukaryotes from yeast to humans and regulates responses to energy limitation (1). Mammalian AMPK, often referred to as the cell’s “fuel gauge” (2), responds to decreases in cellular energy by sensing increased AMP-to-ATP ratios (3, 4). AMPK activation involves AMP binding to the γ-subunit of the kinase complex at the exclusion of ATP, making the catalytic α-subunit a better substrate for activation loop threonine (Thr172) phosphorylation by upstream kinases while interfering with its dephosphorylation by phosphatases. In addition to AMP binding, ADP binding has also been shown to stimulate Thr172 phosphorylation of AMPK and to protect it from dephosphorylation (5, 6). AMPK regulates gene expression and metabolic enzyme activity to maintain energy homeostasis. Important functions of AMPK include activation of energy-generating processes, such as glucose uptake and fatty acid oxidation, as well as inhibition of energy-consuming processes, such as cell growth and proliferation. In accord with these functions, defects in AMPK signaling have been linked to diabetes, obesity, and cancer (7, 8).

In the yeast *Saccharomyces cerevisiae*, Snf1 protein kinase is the ortholog of mammalian AMPK (1, 9). Yeast cells prefer glucose as their carbon and energy source, and one of the key functions of Snf1 is to regulate responses to carbon/energy stress caused by glucose depletion. Once activated, Snf1 promotes the utilization of alternative carbon sources. Mutation of the *SNF1* gene, which encodes the catalytic α-subunit of the Snf1 kinase complex, causes defects in utilization of less-preferred fermentable sugars, such as sucrose or galactose. Snf1 is also essential for respiratory metabolism, and *snf1* mutants fail to grow on nonfermentable carbon sources such as ethanol and glycerol (10, 11).

Like mammalian AMPK, the Snf1 kinase complex is a heterotrimer: in addition to the catalytic α-subunit Snf1, it contains one of three alternate targeting/scaffolding β-subunits (Sip1, Sip2, Gal83) and the stimulatory γ-subunit (Snf4) (9). Any of the three upstream kinases (Sak1, Tos3, Elm1) can activate Snf1 during carbon/energy stress by phosphorylating the conserved Thr210 residue in the activation loop of the Snf1 kinase domain (12–14). When glucose is abundant, Thr210 dephosphorylation (and therefore Snf1 downregulation) involves type 1 protein phosphatase Glc7 in association with regulatory subunits Reg1 and Reg2 (12, 15–17), as well as the type 2A-related phosphatase Sit4 and the type 2C phosphatase Ptc1 (18, 19). The signaling process by which the Snf1 pathway senses changes in glucose/energy availability is not completely understood. *In vitro* experiments show that ADP binding to the γ-subunit Snf4 can protect the catalytic α-subunit Snf1 from Thr210 dephosphorylation and downregulation (20, 21), but an alternative ADP-sensing mechanism has also been proposed, and a Snf4-independent signaling mechanism(s) clearly exists *in vivo* (12, 22, 23).

Snf1 protein kinase is also regulated at the level of subcellular localization (9). The three alternate β-subunits define three isoforms of the kinase and determine their respective localization (24); we will refer to these kinase isoforms as Snf1-Sip1, Snf1-Sip2, and Snf1-Gal83 (25). In the presence of abundant glucose, all three isoforms are cytoplasmic. Upon catalytic activation in response to carbon stress, Snf1-Sip1 localizes to the vacuolar periphery, Snf1-Sip2 remains cytoplasmic, and Snf1-Gal83 is enriched in the nucleus (24–27). The nuclear enrichment of Snf1 contributes to its ability to control the expression of a multitude of genes, not only by regulating transcription activators and repressors but also by modification of chromatin and by effects on the transcription apparatus (24, 28–31).

The nuclear localization of Snf1-Gal83 is regulated at two distinct levels. First, Snf1 must be catalytically activated, otherwise Snf1-Gal83 will be retained in the cytoplasm (25, 27). The existence of a second mechanism becomes unmasked when the *SNF1* gene is deleted: in the *snf1*Δ mutant, Gal83 is cytoplasmic in the presence of abundant glucose but is enriched in the nucleus under carbon stress conditions (24, 25). It has been shown that the principal Snf1-activating kinase Sak1 (formerly Pak1) is necessary for Snf1 nuclear enrichment, but this requirement might simply reflect Sak1’s role in providing sufficient Snf1 catalytic activation, as Sak1 is dispensable for the nuclear enrichment of Gal83 in the absence of Snf1 (25). Evidence suggests a role for the nuclear export receptor Crm1 in the nuclear exclusion of Gal83 during growth on abundant glucose (27). The mechanisms that promote Gal83 nuclear enrichment in response to carbon stress, however, are not completely understood.

The eukaryotic voltage-dependent anion channel (VDAC) proteins, also known as mitochondrial porins, are conserved in evolution and mediate mitochondrial outer membrane permeability to small metabolites, notably ATP, ADP, and AMP (32). As such, VDACs are poised to negatively regulate AMPK by virtue of replenishing the cell with mitochondrially produced ATP. However, our results in yeast reveal the existence of an opposite relationship. *S. cerevisiae* encodes two paralogous VDAC proteins, Por1 and Por2, which are 50% identical in primary structure and localize to the mitochondrial outer membrane (33). At the same time, while Por1 serves as a metabolite channel, Por2 has apparently lost this function; Por2 is also expressed at a lower level than Por1 (33). The *por1*Δ mutation confers a defect in nonfermentable carbon source utilization, whereas the *por2*Δ mutation does not (33). Interestingly, however, overexpression of Por2 can suppress the *por1*Δ mutant for its growth defect on nonfermentable carbon sources, suggesting that Por1 and Por2 can play regulatory roles not directly related to channel function (33). These considerations raised the possibility that Por1 and Por2 play positive regulatory roles in the Snf1 kinase pathway, since this kinase is essential for responses to carbon/energy stress.

Indeed, we previously found that Por1 and Por2 play redundant roles in promoting Snf1 catalytic activation by Thr210 phosphorylation (34). Here, we present evidence that Por1 also promotes Snf1 nuclear localization by a mechanism that is distinct from its role in Snf1 catalytic activation and reflects a role in promoting the nuclear enrichment of the β-subunit Gal83. Our findings expand the positive roles played by Por1/Por2 in carbon/energy stress signaling upstream of Snf1.

## RESULTS

### The *por1*Δ mutation strongly affects transcription activation by LexA-Snf1-G53R.

To better understand the roles of Por1 and Por2 in Snf1 regulation, we used the “shortcut” reporter assay (28). This assay tests for the ability of a hyperactive version of Snf1 with a Gly53-to-Arg substitution fused to the LexA DNA-binding protein (LexA-Snf1-G53R) to activate transcription of a reporter containing LexA operator sequences (*lexAop*) upstream of the *lacZ* gene (*lexAop-lacZ* reporter). Activation of the *lexAop-lacZ* reporter by LexA-Snf1-G53R does not rely on gene-specific transcription factors and occurs by a direct effect on RNA polymerase II holoenzyme (hence the term shortcut) (28). Under glucose-rich conditions, reporter activation by LexA-Snf1-G53R is negligible, but it increases dramatically in response to glucose limitation in a manner that requires both catalytic activation and nuclear localization of the Snf1-G53R moiety (24, 25, 28). As a simple glucose-regulated system, the shortcut reporter assay has served as a valuable tool for examining the mechanisms that control Snf1 catalytic activation and nuclear localization (24, 25, 28). For example, LexA-Snf1-G53R does not activate reporter expression in cells lacking the principal Snf1-activating kinase Sak1 or the nuclear-targeting β-subunit Gal83 (24, 25).

**FIG 1:**
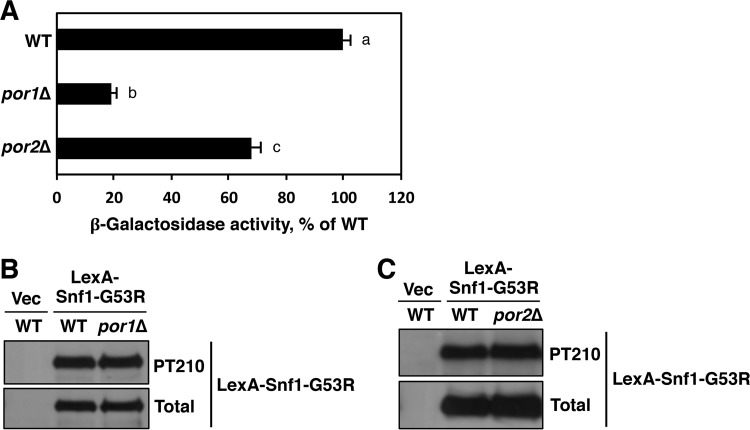
The *por1*Δ mutation strongly affects *lexAop-lacZ* reporter activation by LexA-Snf1-G53R. (A) Strain CTY10-5d (WT) and its *por1*Δ and *por2*Δ mutant derivatives expressing LexA-Snf1-G53R were grown in selective SC medium containing high (2%) glucose to mid-log phase and then shifted for 3 h to an otherwise identical medium containing low (0.05%) glucose. β-Galactosidase activity was assayed in permeabilized cells and measured in Miller units (6 to 9 independent transformants per genotype). The graph shows the data under low-glucose conditions, and they are expressed as the percentage of the wild-type value. Under high-glucose conditions, all values were <3% of the wild-type value under low-glucose conditions. The mean wild-type value in low glucose was 278 Miller units. Error bars indicate standard errors. Statistical analyses were conducted using the two-tailed Student *t* test. Three pairwise tests were performed. The significance level for each test was Bonferroni adjusted from α = 0.05 to α_adj_ = 0.016. All three pairwise *P* values were less than α_adj_ (all *P* < 0.001). The plotted values are labeled with different lowercase letters (a, b, and c) to indicate that they are significantly different from each other. (B and C) Transformants were shifted to 0.05% glucose as described above and tested for Thr210 phosphorylation of the Snf1-G53R moiety (PT210) and total LexA-Snf1-G53R protein levels by immunoblotting. Vec, empty vector control.

Strain CTY10-5D carries a genomically integrated copy of the *lexAop-lacZ* reporter and was previously used for the shortcut assay (28). We therefore expressed LexA-Snf1-G53R in CTY10-5D and its *por1*Δ and *por2*Δ mutant derivatives. Cells were grown to mid-log phase on abundant glucose (2%), and reporter activation was induced by shifting the cells to low glucose (0.05%) for 3 h (28). The *por1*Δ mutant exhibited a substantial transcription activation defect (Fig. 1A). This defect could not be attributed to reduced Thr210 phosphorylation of the Snf1-G53R moiety (Fig. 1B), arguing against a defect in catalytic activation and, hence, suggesting a defect in nuclear localization.

### The *por1*Δ mutation affects the nuclear enrichment of Snf1.

**FIG 2:**
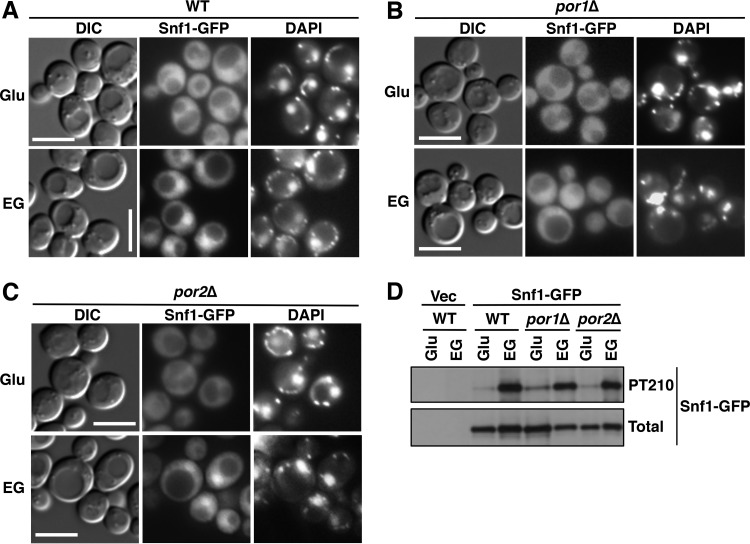
The *por1*Δ mutation affects Snf1 nuclear enrichment. (A to C) Localization of Snf1-GFP was determined by fluorescence microscopy after growth to mid-log phase in selective SC containing 2% glucose (Glu) and after a 20-min shift to an otherwise identical medium containing 3% ethanol and 2% glycerol (EG) instead of glucose. The cells were imaged by differential interference contrast (DIC) microscopy. Nuclei were stained with DAPI. Scale bars, 5 μm. (D) Cells were grown as described above, and the Thr210 phosphorylation state (PT210) and total levels of Snf1-GFP (total) were analyzed by immunoblotting. Vec, empty vector control; WT, wild type.

We next tested for effects of the mutations on Snf1 nuclear localization by using a Snf1-green fluorescent protein (GFP) fusion expressed from the native *SNF1* promoter on a low-copy-number centromeric plasmid. Wild-type and mutant cells were grown on abundant glucose, and the nuclear enrichment of Snf1 was stimulated by shifting the cells to a mixture of ethanol and glycerol (ethanol-glycerol) as the carbon source for 20 min (24, 25). As expected, Snf1-GFP was excluded from the nuclei of glucose-grown wild-type cells (Fig. 2A); Snf1-GFP was similarly excluded from the nuclei of the *por1*Δ and *por2*Δ mutants (Fig. 2B and C). Upon the shift to ethanol-glycerol, Snf1-GFP was enriched in the nucleus in the wild type, as anticipated (24), as well as in the *por2*Δ mutant (Fig. 2A and C). However, no nuclear enrichment was observed in *por1*Δ cells (Fig. 2B), and this defect was not associated with a reduction in activation loop Thr210 phosphorylation of Snf1-GFP (Fig. 2D). Together with the findings from the reporter activation assay, these results strongly suggest that the *por1*Δ mutation affects Snf1 nuclear localization by a mechanism that is independent of its catalytic activation.

### The *por1*Δ mutation affects the nuclear enrichment of Gal83 in the presence and absence of Snf1.

**FIG 3:**
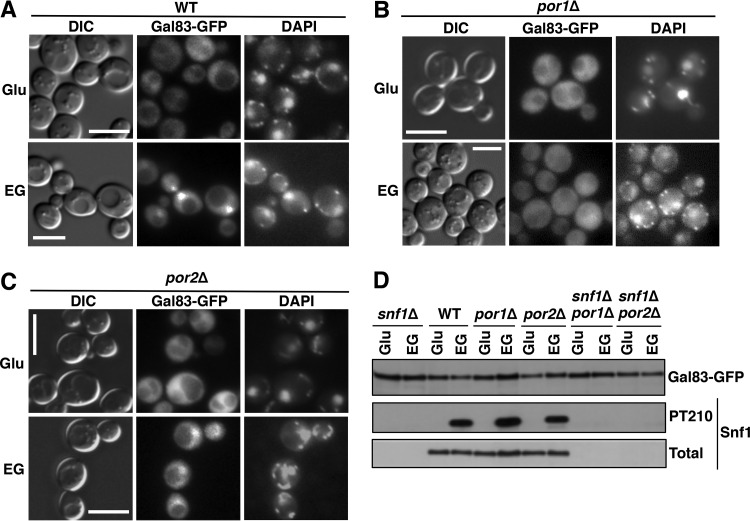
The *por1*Δ mutation affects Gal83 nuclear enrichment. (A to C) Localization of Gal83-GFP was determined by fluorescence microscopy after growth to mid-log phase in selective SC containing 2% glucose (Glu) and then following a 20-min shift to an otherwise identical medium containing 3% ethanol and 2% glycerol (EG) instead of glucose. The cells were imaged by differential interference contrast (DIC) microscopy. Nuclei were stained with DAPI. Scale bars, 5 μm. (D) Cells were grown as described for panels A to C, and Gal83-GFP protein levels as well as Thr210 phosphorylation (PT210) and total levels of Snf1 were analyzed by immunoblotting. WT, wild type.

Nuclear localization of Snf1 depends on Gal83, one of three alternate β-subunits of the Snf1 kinase complex (24, 25). We therefore examined Gal83 localization by using a Gal83-GFP fusion expressed from the native *GAL83* promoter on low-copy-number centromeric plasmid pRT12 (24, 25). During growth on glucose, Gal83-GFP was excluded from the nucleus in all strains tested (Fig. 3A to C). Upon the shift to ethanol-glycerol, Gal83-GFP was enriched in the nuclei of wild-type and *por2*Δ mutant cells (Fig. 3A and C), but no enrichment was observed in the *por1*Δ mutant (Fig. 3B). Immunoblot analysis did not reveal any obvious differences in Gal83-GFP expression or Snf1 Thr210 phosphorylation in the *por1*Δ mutant relative to that in the wild type or *por2*Δ mutant (Fig. 3D).

**FIG 4:**
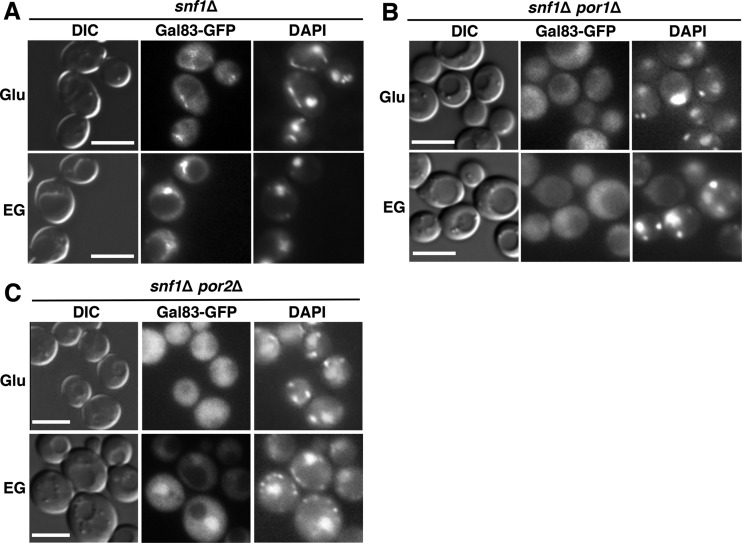
The *por1*Δ mutation affects Gal83 nuclear enrichment in the absence of Snf1. (A to C) Localization of Gal83-GFP was determined by fluorescence microscopy after growth to mid-log phase in selective SC containing 2% glucose (Glu) and then following a 20-min shift to an otherwise identical medium containing a mixture of 3% ethanol and 2% glycerol (EG) instead of glucose. The cells were imaged by differential interference contrast (DIC) microscopy. Nuclei were stained with DAPI. Scale bars, 5 μm.

Previous evidence indicated that Gal83 localization remains regulated in the absence of Snf1: in glucose-grown *snf1*Δ cells, Gal83 is excluded from the nucleus, and it is enriched in the nucleus upon the shift to ethanol-glycerol (24, 25). To address the possible roles of the VDAC proteins in this process, we expressed Gal83-GFP in *snf1*Δ, *snf1*Δ *por1*Δ, and *snf1*Δ *por2*Δ mutant cells (see immunoblotting results in Fig. 3D) and examined its localization during growth on abundant glucose and after a shift to ethanol-glycerol. We observed that just as in the *por1*Δ single mutant, Gal83-GFP was not enriched in the nucleus in the *snf1*Δ *por1*Δ double mutant (Fig. 4B). No such defect was observed in the *snf1*Δ *por2*Δ double mutant (Fig. 4C).

Collectively, these results provide strong evidence that the requirement of Por1 for the nuclear enrichment of Snf1-Gal83 does not reflect its role in Snf1 catalytic activation; instead, it reflects its role in promoting the nuclear enrichment of Gal83.

### Overexpression of Por2 suppresses the *por1*Δ mutant for the Snf1-Gal83 nuclear localization defect.

**FIG 5:**
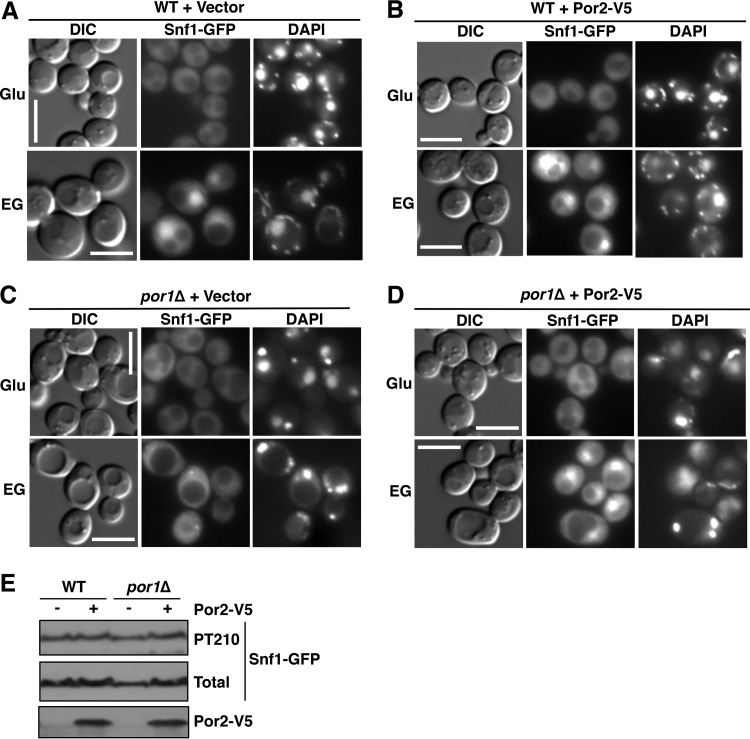
Overexpression of Por2 suppresses the *por1*Δ mutation for the Snf1 nuclear enrichment defect. (A to D) Cells expressing Snf1-GFP and either overexpressing Por2-V5 or carrying the corresponding empty vector were grown to mid-log phase in selective SC medium containing 2% glucose (Glu) and then shifted to an otherwise identical medium containing 3% ethanol and 2% glycerol (EG) instead of glucose. The cells were imaged by differential interference contrast (DIC) microscopy. Nuclei were stained with DAPI. Scale bars, 5 μm. (E) Cells expressing Snf1-GFP and overexpressing Por2-V5 (+) or carrying the corresponding vector (−) were shifted to ethanol-glycerol as described above, and the levels of Snf1-GFP phosphorylated at Thr210 (PT210), total Snf1-GFP, and Por2-V5 were analyzed by immunoblotting. WT, wild type.

**FIG 6:**
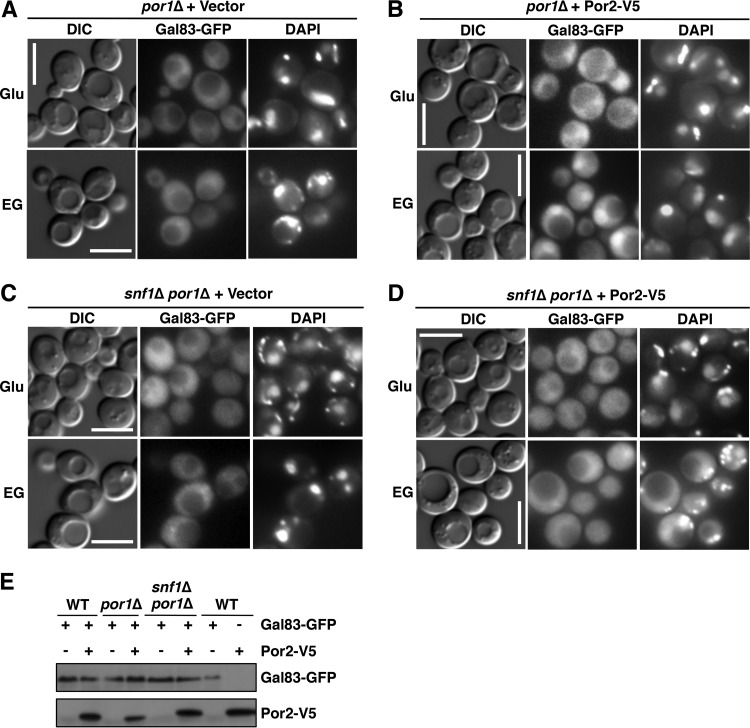
Effects of Por2 overexpression on Gal83 localization. (A to D) Cells expressing Gal83-GFP and either overexpressing Por2-V5 or carrying the corresponding empty vector were grown to mid-log phase in selective SC medium containing 2% glucose (Glu) and then shifted to an otherwise identical medium containing 3% ethanol and 2% glycerol (EG) instead of glucose. The cells were imaged by differential interference contrast (DIC) microscopy. Nuclei were stained with DAPI. Scale bars, 5 μm. (E) Cells expressing Gal83-GFP and overexpressing Por2-V5 (+) or carrying the corresponding vectors (−) were shifted to ethanol-glycerol, as described above, and expression of Gal83-GFP and Por2-V5 was confirmed by immunoblotting. WT, wild type.

The *POR2* gene was first identified as a multicopy suppressor of the *por1*Δ mutant defect during growth on nonfermentable carbon sources (33). We therefore tested whether Por2 overexpression could suppress the *por1*Δ mutant for the Snf1 and Gal83 nuclear enrichment defects. We overexpressed Por2 (as an epitope-tagged Por2-V5 protein) from the yeast *ADH1* promoter on a multicopy plasmid in wild-type and *por1*Δ mutant cells and examined Snf1-GFP localization, as described above. Upon the shift to ethanol-glycerol, Snf1-GFP was enriched in the nuclei of *por1*Δ mutant cells overexpressing Por2-V5, but not in the nuclei of cells carrying the corresponding empty vector (Fig. 5A to D). The compensatory effect of Por2 overexpression was not accompanied by an effect on Snf1 catalytic activation, as evidenced by immunoblotting results for Snf1 activation loop Thr210 phosphorylation (Fig. 5E). Overexpression of Por2-V5 similarly restored the nuclear enrichment of Gal83-GFP in the *por1*Δ mutant (Fig. 6A and B). Thus, these results provide evidence that Por2 overexpression can functionally compensate for the lack of Por1 in promoting the nuclear enrichment of Snf1-Gal83.

We note, however, that overexpression of Por2-V5 did not restore nuclear enrichment of Gal83-GFP in the *snf1*Δ *por1*Δ double mutant (Fig. 6C and D), suggesting that the ability of overexpressed Por2 to make up for the loss of Por1 is Snf1 dependent. Further experiments are required to address the nature of this dependence.

### Overexpression of Por2 suppresses the *por1*Δ mutant defect in transcription activation by LexA-Snf1-G53R in a Gal83-dependent manner.

**FIG 7:**
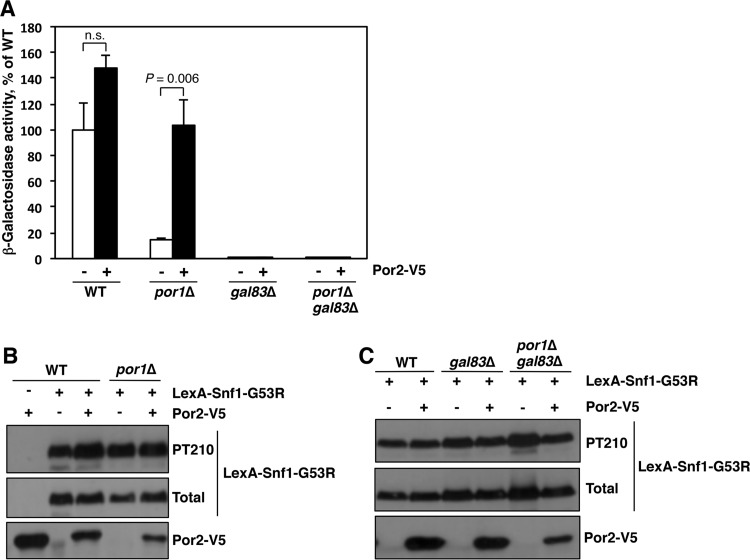
Overexpression of Por2 suppresses the *por1*Δ mutant defect in transcriptional activation by LexA-Snf1-G53R. (A) Reporter strain CTY10-5d (WT) and its mutant derivatives expressing LexA-Snf1-G53R and either overexpressing Por2-V5 (+) or carrying the corresponding empty vector (−) were grown in selective SC containing high (2%) glucose to mid-log phase and then shifted for 3 h to an otherwise identical medium containing low (0.05%) glucose. β-Galactosidase activity was assayed in permeabilized cells and measured in Miller units (4 or 5 independent transformants per genotype/plasmid combination). Shown are the values under low-glucose conditions expressed as a percentage of the mean value for the wild type plus vector (347 Miller units); in high glucose, all values were <3% of this reference value. Error bars indicate standard errors. Statistical analyses were conducted using the two-tailed Student *t* test. Four tests were performed. The significance level was Bonferroni adjusted from α = 0.05 to α_adj_ = 0.012. The graph shows the results for two of these tests. n.s., not significant (*P* = 0.071). In addition, the value for *por1*Δ without Por2-V5 was significantly different from that for the wild type without Por2-V5 (*P* = 0.009). All values for *gal83*Δ and *gal83*Δ *por1*Δ with or without Por2-V5 were small (<1% of the reference value for the wild type without Por2-V5); as a group, these small values were significantly different even from the next smallest value for *por1*Δ without Por2-V5 (*P* < 0.001). (B and C) Transformants were shifted to 0.05% glucose as described for panel A and tested for Thr210 phosphorylation of the Snf1-G53R moiety (PT210), total LexA-Snf1-G53R protein levels, and Por2-V5 expression by immunoblotting.

We also tested for effects of Por2 overexpression on *lexAop-lacZ* reporter activation by LexA-Snf1-G53R. Reporter strain CTY10-5D and its *por1*Δ mutant derivative expressing LexA-Snf1-G53R were transformed with a plasmid overexpressing Por2-V5 or with the corresponding empty vector. Transformants were grown on abundant glucose to mid-log phase, and reporter activation was induced by shifting the cells to low glucose for 3 h (28). Overexpression of Por2-V5 in *por1*Δ cells restored reporter activation to a level comparable to that in the wild-type cells (Fig. 7A). This effect could not be attributed to an effect on the expression of the LexA-Snf1-G53R fusion protein or to Thr210 phosphorylation of its kinase moiety (Fig. 7B).

The ability of LexA-Snf1-G53R to activate transcription of the *lexAop-lacZ* reporter depends on Gal83 (24). If Por2 overexpression suppresses the *por1*Δ defect in reporter activation by restoring Gal83 nuclear localization, then the requirement for Gal83 should not be bypassed. We therefore examined the effects of Por2 overexpression on reporter activation in *gal83*Δ and *por1*Δ *gal83*Δ mutants. Indeed, LexA-Snf1-G53R failed to activate transcription in these mutants regardless of overexpressed Por2-V5 (Fig. 7A and C). Thus, these reporter activation results further support the idea that Por1 and Por2, when the latter is overexpressed, promote Snf1 nuclear localization via a Gal83-mediated mechanism and not through effects on some other β-subunit.

### Hyperactive Snf1-G53R restores the ability of the *por1*Δ mutant to grow on nonfermentable carbon sources in a Gal83-dependent manner.

**FIG 8:**
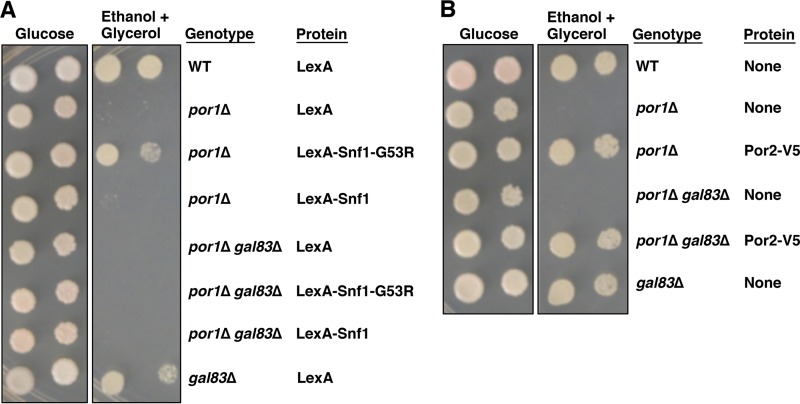
Requirement of Gal83 for suppression of the *por1*Δ mutation. (A) Hyperactive LexA-Snf1-G53R ameliorates the effects of the *por1*Δ mutation in a Gal83-dependent manner. Transformants were grown in selective SC containing 2% glucose to mid-log phase and then washed, and serial dilutions were spotted onto selective SC plates with either 2% glucose or a mixture of 3% ethanol and 2% glycerol as carbon sources. The plates were incubated at 30°C for 2 and 4 days, respectively. (B) Overexpression of Por2 ameliorates the effects of the *por1*Δ mutation, independent of Gal83. Transformants were grown as described for panel A. WT, wild type.

In the course of our experiments, we noted that expression of hyperactive LexA-Snf1-G53R suppressed the *por1*Δ mutation for defective growth on ethanol-glycerol (Fig. 8A). This result might indicate that the primary cause of the *por1*Δ mutant growth defect is reduction of overall Snf1 kinase activity in the cell. However, our results strongly suggest that the *por1*Δ mutation does not lead to a defect in Snf1 catalytic activation. We therefore hypothesized that the ability of hyperactive LexA-Snf1-G53R to suppress the *por1*Δ mutation reflects not an overall boost of cellular Snf1 activity but specifically an increase of Snf1 activity in the nucleus. If so, the ability of LexA-Snf1-G53R to suppress *por1*Δ would be expected to depend on Gal83. Indeed, expression of LexA-Snf1-G53R failed to restore growth of a *por1*Δ *gal83*Δ double mutant on ethanol-glycerol (Fig. 8A). Thus, one of the reasons for the *por1*Δ mutant’s growth defect on nonfermentable carbon sources is likely to be a reduction in nuclear Snf1 activity. We note, however, that this cannot be the only cause, since the *gal83*Δ mutation, which affects Snf1 nuclear localization, does not abolish growth on ethanol-glycerol (35) (Fig. 8A). We believe that Por1 plays regulatory roles in at least two distinct processes that are coessential for growth on nonfermentable carbon sources. One process is Gal83-dependent nuclear enrichment of Snf1. The other process is Gal83 independent, since overexpression of Por2 can still suppress the growth defect of the *por1*Δ *gal83*Δ double mutant (Fig. 8B). In other words, the growth defect of the *por1*Δ mutant on ethanol-glycerol could be explained as a synergy of two defects, and ameliorating either one of these defects is sufficient to restore growth.

## DISCUSSION

The *POR1* and *POR2* genes of *S. cerevisiae* represent a pair of paralogous genes that arose from an ancient whole-genome duplication event (36). Interestingly, while most duplicate gene copies have since been lost, many of the remaining gene pairs function in energy metabolism and its regulation (37, 38). This, together with the freedom of the paralogous partners to acquire a degree of specialization, may have been beneficial for the ability of *S. cerevisiae* to adapt to the quality of the available carbon/energy source. An interesting example of such a paralogous gene pair is *SNF3* and *RGT2*. Although Snf3 and Rgt2 are glucose transporter homologs, they have lost their transporter activity and instead function as high-affinity and low-affinity glucose sensors, respectively, that regulate gene expression in response to extracellular glucose (39, 40). By analogy, the retention of the *POR2* gene, whose product localizes to the mitochondrial outer membrane but does not appear to have channel function (33), raises the possibility that Por2 and, by extension, Por1 serve as metabolic sensors to regulate responses to a potentially large number of cues.

While the notion that Por1 and Por2 could serve as sensors is suggested by the apparent lack of channel function in Por2, the clues as to what they might sense are suggested by what Por1 transports. The major function of eukaryotic VDACs, including yeast Por1, is to mediate mitochondrial outer membrane permeability to small metabolites, notably adenine nucleotides, and it has been shown that ATP, ADP, and AMP can also bind to VDAC (41).

Snf1 protein kinase would be an obvious beneficiary of such a metabolite-sensing function of Por1 and Por2. As with mammalian AMPK, Snf1 activity correlates with the cellular AMP-to-ATP ratios (42). However, unlike AMPK, which uses its γ-subunit to sense adenine nucleotides, the yeast Snf1 kinase complex does not appear to utilize its respective γ-subunit Snf4 for adenine nucleotide sensing *in vivo* (12, 22, 23). We previously showed that Por1 and Por2 play redundant roles in the positive control of Snf1 catalytic activation by activation loop Thr210 phosphorylation (34). Here, we present evidence that Por1 also plays an important role in promoting the nuclear enrichment of the Snf1-Gal83 isoform of the kinase in response to carbon stress.

Our results suggest that the new role played by Por1 in Snf1-Gal83 nuclear enrichment is separable from its role in catalytic activation of the kinase, and rather it reflects a role in regulating the nuclear localization of Gal83 itself. First, the *por1*Δ mutation alone does not affect Thr210 phosphorylation of Snf1. Second, Gal83 fails to enrich in the nucleus of the *por1*Δ *snf1*Δ double mutant. To our knowledge, Por1 seems to represent the first protein identified to date with a role in promoting Gal83 nuclear enrichment.

In addition, the role played by Por1 in Snf1-Gal83 nuclear enrichment appears to be separable from its role in mitochondrial outer membrane permeability and its direct contribution to mitochondrial respiration. First, the nuclear enrichment defect of the *por1*Δ mutant can be suppressed by overexpression of the non-channel-forming paralog Por2. Second, Gal83 can be enriched in the nucleus of the *snf1*Δ mutant (24, 25), despite the profound defects of this mutant in respiratory metabolism and nonfermentable carbon source utilization (9). Thus, Por1 appears to play a purely regulatory role, supporting the idea that it could function as an upstream sensor in this process.

The signaling mechanisms that regulate Gal83 nuclear localization in response to the carbon source are not known. It was previously shown that elimination of all three enzymes that can phosphorylate glucose (Hxk1, Hxk2, and Glk1) results in constitutive (glucose-insensitive) nuclear enrichment of Snf1-Gal83 (24). This suggested that Snf1-Gal83 nuclear localization is blocked not by extracellular glucose but rather by a glucose metabolite. The same study also showed that this metabolite is likely to be glucose-6-phosphate, since treating the cells with the glucose analog 2-deoxyglucose is sufficient to cause this block (24). However, subsequent work showed that 2-deoxyglucose inhibits Snf1-Gal83 nuclear enrichment by affecting Snf1 kinase activity rather than by affecting the nuclear localization of Gal83 itself (27). Thus, if Por1 mediates a glucose-related signal affecting Gal83, it does not seem likely that this signal is glucose-6-phosphate; however, it remains possible that Por1 and Por2 mediate a glucose-6-phosphate signal in their capacity as regulators of Snf1 catalytic activation. We do not currently know what signal Por1 senses to regulate the nuclear localization of Gal83, nor do we know how Por1—a mitochondrial outer membrane protein—could affect the function of the nuclear transport process. Further studies are necessary to address these questions.

It is important to note that the regulatory role of Por1 in the carbon stress response (i.e., the role that can be replaced by Por2 overexpression) is not limited to promoting the nuclear enrichment of Snf1-Gal83. Indeed, our results indicate that overexpression of Por2 can still suppress the *por1*Δ *gal83*Δ double mutant for its growth defect on nonfermentable carbon sources. This clearly suggests that VDAC proteins have the capacity to play regulatory roles in multiple pathways.

Because Snf1/AMPK and VDAC proteins are conserved in evolution, our results in yeast may have implications for AMPK regulation in other eukaryotes, including humans. Since VDACs release mitochondrially produced ATP to the cytoplasm, they would be predicted to contribute to the negative control of AMPK. Our experiments in yeast indicate the existence of an opposite effect, which occurs not only at the level of catalytic activation but also at the level of nuclear enrichment of the kinase. This suggests that VDAC proteins could play complex roles in AMPK regulation in other eukaryotes.

## MATERIALS AND METHODS

### Yeast strains and growth conditions.

The *S. cerevisiae* strains used in this study are listed in Table 1. With the exception of the *lexAop-lacZ* reporter strain CTY10-5d (R. Sternglanz, SUNY, Stony Brook, NY) (43) and its mutant derivatives, the strains were in the W303 genetic background and were descendants of strains W303-1A (*MAT***a** *ade2-1 can1-100 his3-11,15 leu2-3,112 trp1-1 ura3-1*) and W303-1B (*MAT*α *ade2-1 can1-100 his3-11,15 leu2-3,112 trp1-1 ura3-1*) (44). Construction of W303 derivatives carrying the *por1*Δ::*KanMX6* and *por2*Δ::*KanMX6* alleles has been described previously (34). To generate *snf1Δ*::*KanMX6* and *gal83Δ*::*KanMX6* derivatives, the marker sequences were amplified by PCR with primers flanking the corresponding open reading frames. The mutant alleles were first introduced into a wild-type W303 diploid strain by transformation; all transformations were performed using standard methods (45). Haploid mutants were then recovered from the heterozygous diploids by tetrad analysis. Combinatorial W303 mutants were generated by genetic crossing and tetrad analysis. The *por1Δ*::*KanMX6*, *por2Δ*::*KanMX6*, and *gal83Δ*::*KanMX6* mutant derivatives of the reporter strain CTY10-5D (YSK1271, YSK1274, and AMS302, respectively) were generated similarly, except that the mutant alleles were introduced directly into CTY10-5d (*MAT***a*** gal4 gal80 URA3*::*lexAop-lacZ his3 leu2 ade2 trp1*). The *por1Δ gal83Δ* double mutant *lexAop-lacZ* reporter strain (AMS293) was constructed by tetrad analysis of a cross between appropriate mutant derivatives of CTY10-5D and W303. The *por1*Δ, *por2*Δ, *snf1*Δ, and *gal83*Δ knockout genotypes of all mutants were confirmed by PCR analysis of genomic DNA.

**TABLE 1:** *S. cerevisiae* strains

Strain	Genotype	Source
CTY10-5d	*MAT***a*** gal4 gal80 URA3*::*lexAop-lacZ his3 leu2 ade2 trp1*	R. Sternglanz
YSK1271	*MAT***a*** gal4 gal80 URA3*::*lexAop-lacZ his3 leu2 ade2 trp1 por1*Δ::*KanMX6*	This study
YSK1274	*MAT***a*** gal4 gal80 URA3*::*lexAop-lacZ his3 leu2 ade2 trp1 por2*Δ::*KanMX6*	This study
AMS302	*MAT***a*** gal4 gal80 URA3*::*lexAop-lacZ his3 leu2 ade2 trp1 gal83*Δ::*KanMX6*	This study
AMS293	*MAT***a*** URA3*::*lexAop-lacZ his3 leu2 ade2 trp1 por1*Δ::*KanMX6 gal83*Δ::*KanMX6* Gal^−^	This study
MMY35	*MAT***a** *ade2-1 can1-100 his3-11,15 leu2-3,112 trp1-1 ura3-1*	This lab
YSK1279	*MAT***a*** ade2-1 can1-100 his3-11,15 leu2-3,112 trp1-1 ura3-1 por1*Δ::*KanMX6*	This lab
YSK1283	*MAT***a*** ade2-1 can1-100 his3-11,15 leu2-3,112 trp1-1 ura3-1 por2*Δ::*KanMX6*	This lab
AMS125	*MAT***a*** ade2-1 can1-100 his3-11,15 leu2-3,112 trp1-1 ura3-1 snf1*Δ::*KanMX6*	This study
AMS135	*MAT***a*** ade2-1 can1-100 his3-11,15 leu2-3,112 trp1-1 ura3-1 snf1*Δ::*KanMX6 por1*Δ::*KanMX6*	This study
AMS140	*MAT***a*** ade2-1 can1-100 his3-11,15 leu2-3,112 trp1-1 ura3-1 snf1*Δ::*KanMX6 por2*Δ::*KanMX6*	This study
AMS108	*MAT***a*** ade2-1 can1-100 his3-11,15 leu2-3,112 trp1-1 ura3-1 gal83*Δ::*KanMX6*	This study
AMS298	*MAT***a*** ade2-1 can1-100 his3-11,15 leu2-3,112 trp1-1 ura3-1 por1*Δ::*KanMX6 gal83*Δ::*KanMX6*	This study

Rich medium was yeast extract-peptone (YEP) supplemented with extra tryptophan (40 mg/liter) and adenine (20 mg/liter); synthetic complete (SC) medium lacking appropriate supplements was used to select for plasmids (45). Unless indicated otherwise, the media contained 2% glucose, and cells were grown at 30°C.

### Plasmids.

Plasmids pIT469 and pRJ216 (28) express LexA-Snf1 and LexA-Snf1-G53R, respectively, from the yeast *ADH1* promoter of multicopy vector pEG202 (46). The low-copy-number *CEN-HIS3* plasmid pRT12 (24) expresses a C-terminal GFP-tagged Gal83 (Gal83-GFP) protein from the native *GAL83* promoter in vector pRS313 (47). The low-copy-number *CEN-URA3* plasmid pSnf1-GFP expresses a C-terminal GFP-tagged Snf1 (Snf1-GFP) protein from the native *SNF1* promoter and was derived from pSM14 (48). The multicopy vector pSK71 provides expression from the yeast *ADH1* promoter and was constructed by deleting the LexA-coding sequence from pBTM116 (49). pAMS10 expresses a C-terminal V5 epitope-tagged Por2 (Por2-V5) from vector pSK71.

### Assays of *lexAop-lacZ* reporter activation by LexA-Snf1-G53R.

Cells of strains carrying the integrated *lexAop-lacZ* reporter and expressing LexA-Snf1-G53R were grown in appropriate selective SC medium containing high (2%) glucose to mid-log phase and then shifted for 3 h to an otherwise identical medium containing low (0.05%) glucose. Assays of β-galactosidase activity were performed in permeabilized cells and measured in Miller units as described previously (28).

### Immunoblot analysis.

Cells were grown under conditions specified in the descriptions of experimental results. Protein extracts were prepared by the boiling/alkaline treatment method as described previously (50) and analyzed by immunoblotting. The LexA-Snf1-G53R fusion protein was detected with anti-LexA antibody (Millipore). The Snf1-GFP and Gal83-GFP fusion proteins were detected with anti-GFP antibody (Roche). The epitope-tagged Por2-V5 protein was detected with V5 tag antibody (Thermo Scientific). The endogenous Snf1 protein was detected with anti-polyhistidine antibody H1029 (Sigma-Aldrich), which strongly recognizes Snf1 due to the presence of a natural stretch of 13 consecutive histidines near its N terminus (amino acids 18 to 30) (50). The Thr210 phosphorylation state of Snf1, LexA-Snf1-G53R, and Snf1-GFP was analyzed using anti-phospho-Thr172-AMPK antibody (Cell Signaling), which strongly recognizes the Thr210-phosphorylated form of yeast Snf1 (50). Signals were detected by enhanced chemiluminescence using the Pierce ECL2 or ECL systems (Thermo Scientific).

### Fluorescence microscopy.

Snf1-GFP and Gal83-GFP were expressed in wild-type and mutant cells isogenic to W303-1A. For studies of glucose-regulated localization of these proteins, we followed a protocol published previously (24). Briefly, cells were grown to mid-log phase in appropriate selective SC medium containing 2% glucose and then shifted to otherwise identical medium containing 3% ethanol and 2% glycerol instead of glucose for 20 min (24). Nuclei were stained by adding 10 μl of an 0.8-μg/ml solution of 4′,6-diamidino-2-phenylindole (DAPI) to 1 ml of cell culture and incubating for 5 min at 30°C. The cells were then collected by brief centrifugation, and the DAPI and GFP signals were examined using a Nikon Eclipse 80*i* fluorescence microscope, a CoolSNAP HQ^2^ camera (Photometrics), and NIS-Elements BR 3.01 software.
